# Next-generation molecular imaging of cardiac fibrosis

**DOI:** 10.1007/s10554-026-03682-0

**Published:** 2026-03-21

**Authors:** Martin Ezeani, Marc R. Dweck, Neil J. Craig

**Affiliations:** 1 Department of Biomedical Research and Translational Medicine, Masonic Medical Research Institute, 2150 Bleecker Street, Utica, NY USA; 2 British Heart Foundation Centre of Research Excellence, Centre for Cardiovascular Sciences, University of Edinburgh, Chancellor’s Building, 49 Little France, Edinburgh, Crescent EH16 4SB UK

**Keywords:** Fibrosis, Extracellular matrix, Molecular imaging, Cardiovascular diseases, Diagnosis, Therapeutic monitoring

## Abstract

Cardiac fibrosis is a complex, dynamic pathophysiological process seen in virtually all forms of cardiovascular diseases. However, most current clinical definitions remain limited to static, anatomical constructs. Here we reconceptualise myocardial fibrosis as an active process, driven by transitions in fibroblast states, shaped by multicellular circuits and intrinsically embedded within a spatiotemporal matrix of extracellular remodelling. We present next-generation molecular imaging as a transformative platform to interrogate not only fibrotic burden but also the pathobiological mechanisms underlying disease. Leveraging insights from single-cell and spatial ‘omics, we describe a continuum of fibrotic phenotypes and introduce the concept of matreotypes: molecular fingerprints of extracellular matrix state and function. Advances in tracer development now allow in vivo mapping of key fibrotic mechanisms, including fibroblast activation, matrix proteolysis, collagen cross-linking, and epigenetic remodelling. We challenge the single-target paradigm and advocate for multiplex and activatable probes that capture complex disease biology. This approach integrates spatial, temporal, and systems-level biology to enable dynamic phenotyping and risk stratification. Finally, we propose a closed-loop framework combining imaging, ‘omics, and computational modelling to drive discovery with the aim to improve clinical decision-making, and precision cardiovascular care.

## Introduction

**Fig. 1:**
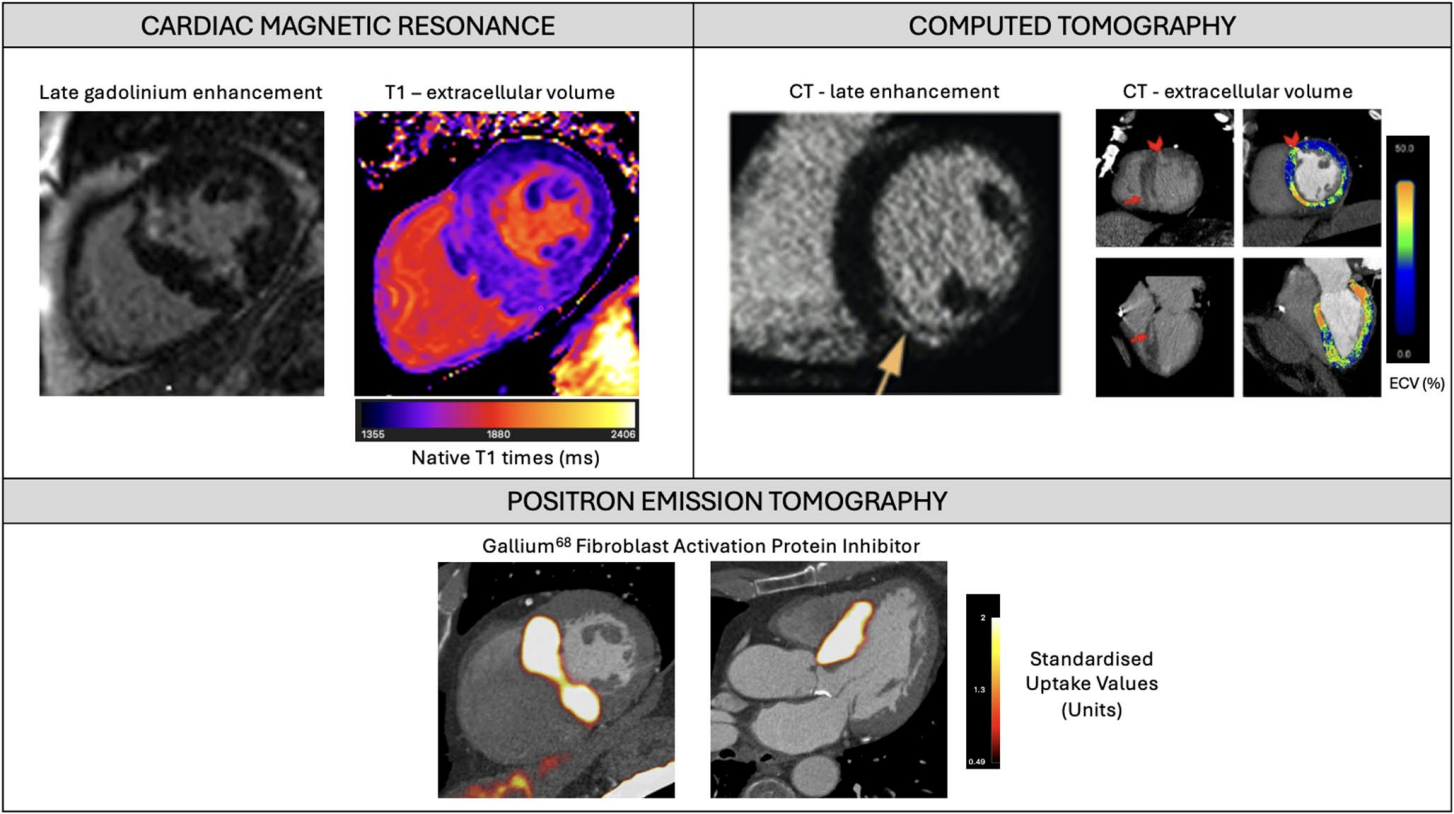
Existing and novel imaging approaches for measuring cardiac fibrosis. The current gold standard methods provide anatomical and physiological information (Upper Left). Cardiac magnetic resonance imaging can detect established replacement and diffuse interstitial fibrosis using surrogate parameters. Novel computed tomography markers have also recently developed which may also be able to detect established fibrosis but similarly to magnetic resonance, is only able to detect expansion of the extracellular space as a surrogate marker for fibrosis. By contrast, molecular imaging is able to detect myocardial fibrosis activity by detecting cellular markers specific for active fibrosis. One example is gallium-68 fibroblast activation protein inhibitor which binds to fibroblast activation protein, expressed on the cell surface of activated fibroblasts

Myocardial fibrosis is the irreversible consequence of chronic injury or cell loss, and is the final common pathophysiological pathway of numerous cardiac diseases. Late gadolinium enhancement on cardiac magnetic resonance imaging represents the current gold-standard non-invasive method for fibrosis assessment. This technique can identify the presence or absence of replacement fibrosis, and can delineate between subendocardial infarct-related fibrosis and midwall non-ischaemic fibrosis (Fig. 1) [1, 2]. However, late gadolinium enhancement is not specific for fibrosis, instead measuring extracellular space expansion as a surrogate, and is unable to provide insights into disease activity and the potential to improve with therapy. Molecular imaging (Fig. 1) and bioinformatics technologies including ribonucleic-acid (RNA) sequencing and spatial transcriptomics have provided insights to the dynamic process of fibrosis development which involves complex relationships between fibroblasts, immune cells, endothelial cells, and the extracellular matrix [3–5]. In this review, we outline the current understanding of myocardial fibrosis, and how integrating advanced molecular imaging approaches and bioinformatics technologies may yield important novel data to inform early disease detection, identification of future and existing therapeutics, stratification of risk, and ultimately improve personalised care.

Fibroblasts, the key effector cell of fibrogenesis, exist in distinct phenotypic states. These include quiescent, activated, inflammatory and senescent fibroblasts that evolve in response to biomechanical stress, inflammation, metabolic alterations, and ageing [6, 7]. Established fibrosis develops through fibroblast activation and collagen cross-linking to result in excessive extracellular matrix deposition [8]. In the heart, fibroblasts detect and respond to environmental changes or pathological stimuli from multiple sources, such as the extracellular matrix, surrounding cells, and soluble mediators via circulating cytokines, hormones, chemokines, and metabolites [9]. Cardiac fibroblast activation is partly mediated by transforming growth factor-β (TGF-β). Canonical TGF-β signalling leads to phosphorylation and nuclear import of SMAD (Sma and Mad Drosophila homologs) transcription factors, which bind to regulatory elements in a variety of pro-fibrotic genes, such as COL1A1 (collagen type I alpha 1 chain), COL3A1 (collagen type III alpha 1 chain), FN1 (fibronectin 1), PAI-1 (plasminogen activator inhibitor-1 (gene symbol: *SERPINE1*)), and CTGF (connective tissue growth factor), thereby driving extracellular matrix deposition and fibrosis. Fibroblast activation and the downstream effects are also orchestrated by a tightly regulated network of transcription factors, chromatin modifiers (e.g., bromodomain-containing protein 4 (BRD4)) and cytokines [10]. Matrix stiffening from lysyl oxidase (LOX)-mediated cross-linking alters mechano-transduction, impairs diastolic myocardial relaxation, and perpetuates fibroblast activation via integrin-mediated signaling [11]. This feedback loop where fibrosis fuels dysfunction and the dysfunction begets further adverse remodelling illustrates the complexity of fibrosis development.

In order to diagnose and treat myocardial fibrosis effectively, we must consider spatial and temporal dynamics, cell–to-cell communication, and regulatory feedback loops. Molecular imaging can identify early, phenotype-specific biological activity, such as fibroblast activation (via fibroblast activation protein (FAP)), matrix metalloproteinase (MMP) activity, or inflammatory cell recruitment (via CXCR4 or CCR2), before irreversible structural changes occur [12, 13]. This technology has the potential to understand cellular pathways driving fibrosis, particularly when combined with novel bioinformatics technologies such as single cell and single nuclei RNA sequencing or spatial transcriptomics.

Just as the field of oncology considers histological, molecular and genetic classifications of disease, variable fibrosis phenotypes exist. These "matreotypes," defined as the composition and modification of extracellular matrix proteins associated with or caused by a disease phenotype, can inform both prognosis and therapeutic strategy [14]. Multiparametric molecular imaging tools to visualise matreotypes and combine perfusion, inflammation and fibrosis assessments can provide a comprehensive assessment of myocardial health, with the potential to inform personalised and precision medicine.

## The cellular and molecular landscape of cardiac fibrosis

Cardiac fibrosis arises not from a singular cell fate or injury response, but from a mosaic of fibroblast phenotypes shaped by local myocardial cues [8, 15]. For example following acute myocardial infarction, fibroblasts in the infarct border zone encounter damage-associated molecular patterns, cytokines, and hypoxia, driving a myofibroblast phenotype that forms dense scar tissue. By contrast, fibroblasts in remote regions remain quiescent or adopt pro-remodelling roles, contributing gradually to long-term extracellular matrix remodelling. Inflammatory myofibroblasts emerge from exposure to immune cell-derived cytokines (e.g., IL-6, TGF-β). In diabetic cardiomyopathy, high glucose or altered fatty acid oxidation induces metabolically reprogrammed fibroblasts that promote extracellular matrix cross-linking and stiffness independent of inflammation. Fibroblasts have the potential to adopt roles from matrix-producing effector cells to inflammatory amplifiers and paracrine regulators, depending on the nature and duration of stress exposure [16, 17]. Recent single-cell RNA sequencing and spatial transcriptomics studies have defined multiple fibroblast subpopulations with distinct transcriptional profiles and spatial niches [4, 10, 18]. Activated fibroblasts expressing periostin (Postn), fibroblast activation protein, and extracellular matrix-modulating enzymes (e.g., Lox, Mmp2) dominate in sites of acute injury, whereas Sfrp2-positive fibroblasts are associated with fibrosis resolution [7]. In parallel, myeloid cells, endothelial cells, and cardiomyocytes engage in reciprocal signalling with fibroblasts, forming multicellular fibrotic circuits that are increasingly recognised as therapeutic targets [19].

The accumulation of structural proteins including collagen I and III results in remodelling and expansion of the extracellular matrix in cardiac fibrosis of all aetiologies. However, this process is influenced by enzymes, matricellular proteins, growth factors, and cross-linkers which form distinct micro-environments that vary with the underlying disease, across disease stages, and affected region of the heart [20, 21]. This is reflected in the concept of a ‘matreotype’, a disease-specific extracellular matrix signature that reflects the underlying cellular and molecular context [22]. Every cell type produces its own matreotype, reflective of cellular identity and physiological status. Molecular imaging of a specific matreotype would therefore allow imaging of a specific disease signature, acting as a biomarker and aiding early diagnosis, prognosis and response to therapy.

Early extracellular matrix remodelling is marked by increased MMP2 and MMP9 activity, which cleaves basement membrane components like collagen IV, generating extracellular matrix fragments that signal to neighbouring cells [23, 24]. In contrast, lysyl oxidase (LOX)-mediated collagen cross-linking defines a late-stage, stiff extracellular matrix matreotype associated with diastolic dysfunction and poor prognosis [25–27]. Additional matreotypes, including proteoglycans (e.g., versican), matricellular proteins (e.g., Postn), secreted protein acidic and rich in cysteine, and matrix-bound cytokines, have been characterised by proteomics and single-cell approaches. Imaging these matreotypes is increasingly feasible through novel positron emission tomography probes targeting collagen-binding peptides (e.g., T-peptide), fibroblast activation protein, and MMP-cleaved collagen IV reporters [28–31]. Genomics-guided tracer design may further enable targeting of matrisome components predictive of disease stage, reversibility, or therapy response. Mapping the matreotypes in vivo has the potential to accurately and specifically phenotype extracellular matrix remodeling, providing insights into biological activity and importantly being able to differentiate between irreversible and potentially regenerative fibrosis.

## Current imaging of myocardial fibrosis

Current imaging approaches for myocardial fibrosis rely upon extracellular space expansion as a surrogate marker but are non-specific. For example, late gadolinium enhancement after acute myocardial infarction may reflect oedema or necrosis rather than fibrosis. Molecular imaging approaches may be more specific for fibrosis depending on the particular radiotracer, and when combined with structural imaging can provide a comprehensive evaluation of active fibrosis and functional consequences for a particular patient. Technological advancements may facilitate multi-tracer administration, for the first time enabling the imaging of dynamic and complex fibrosis pathways in one scan.

Spatial distribution of fibrosis may reflect different pathophysiology and as a result can predict a clinical trajectory. For example, subepicardial fibrosis is suggestive of myocarditis whereas midwall fibrosis is more likely to represent dilated cardiomyopathy [32, 33]. The timing of fibrosis deposition, whether transient, progressive, or persistent can also influence outcomes such as risk of arrhythmia or systolic and diastolic dysfunction. For example, interstitial fibrosis in the interventricular septum has been linked to greater arrhythmogenic potential than similar involvement of the lateral wall [34].

Fibrosis also follows a temporal trajectory, progressing from acute injury through fibroblast activation and matrix deposition, and ultimately towards either resolution or irreversible scarring. Imaging using sequential, stage-specific tracers, enables dynamic visualisation of fibrosis [35]. These temporally sensitive measurements are particularly useful in assessing therapeutic efficacy [36–38]. For instance, successful anti-fibrotic interventions may suppress early extracellular matrix signals (e.g. MMPs, collagen turnover) long before replacement fibrosis or diastolic and systolic dysfunction develops.

## Novel imaging targets for myocardial fibrosis

### Imaging MMP2-proteolized collagen IV for early and late extracellular matrix remodelling

In normal tissue homeostasis, collagen turnover is tightly regulated by matrix metalloproteinases (MMPs), a family of zinc- and calcium-dependent endopeptidases that degrade extracellular matrix proteins in a cell- and context-specific manner [11]. However in cardiac disease, upregulation of MMP2 and MMP9 leads to dysregulated collagen turnover and excessive extracellular matrix remodelling contributing to pathological fibrosis [11]. MMP2-mediated proteolysis exposes cryptic neoepitopes, bioactive sites that are normally buried within the triple-helical domain of collagen IV [24]. These sites mediate cell migration and angiogenesis, but can also serve as molecular targets [24].

**Fig. 2:**
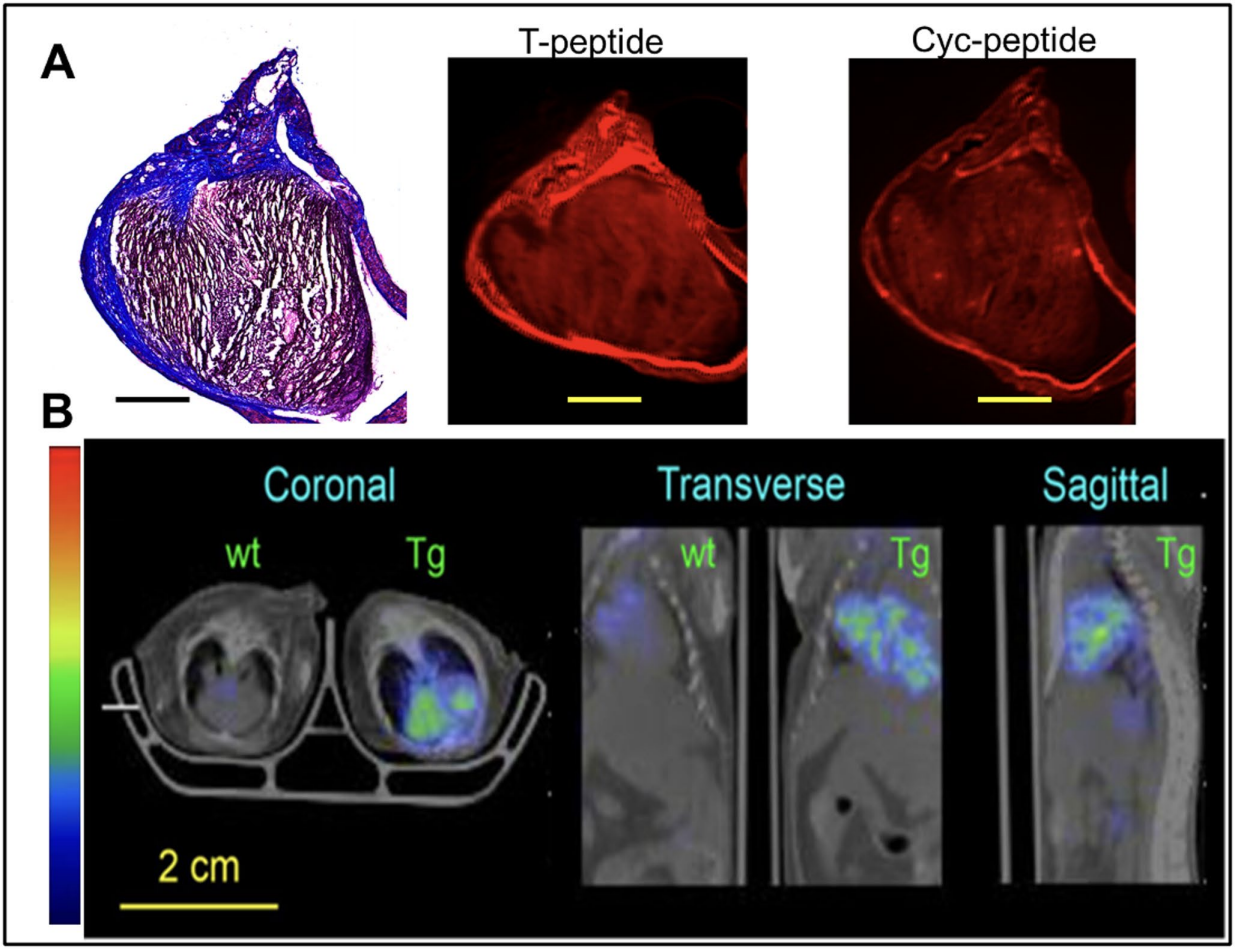
T-peptide positron emission tomography tracer detecting proteolysed Collagen IV for identifying diffuse fibrosis. (**A**) Traditional Mason’s trichrome staining correlated with T-peptide and Cyc-peptide probes for detecting epi-pericardial fibrosis in atrial cardiomyopathy. (**B**) PET/CT scans showed a substantial increase in ^64^Cu-T-peptide-MeCOSar binding in the fibrotic hearts of aged transgenic β2-AR mice with heart failure throughout the entire 45-min acquisition period, compared to wild-type controls. PET/CT scans were obtained following intravenous administration of ^64^Cu-T-peptide-MeCOSar (0.25 mg/kg; 18 ± 2.7 MBq) to the mice. Colour scale represents PET radiotracer uptake with red corresponding to the highest activity and blue to the lowest activity

A heptapeptide termed T-peptide with the sequence TLTYTWS has been identified which exhibits high affinity and selectivity for MMP2-cleaved collagen IV (Table 1) [23]. Initially applied in cancer models, T-peptide was found to accumulate in angiogenic tissue and to inhibit neovascularisation [23]. In particular, enrichment of this processed collagen IV has been identified in the myocardium of patients with dilated cardiomyopathy [39]. T-peptide-based imaging may offer a real-time readout of extracellular matrix degradation. In a mouse model of β2-adrenergic receptor–induced heart failure with atrial cardiomyopathy and heart failure, radiolabelled T-peptide selectively localised to areas of extracellular matrix expansion, correlating with histological remodelling and fibroblast infiltration [30, 31, 40, 41]. These findings suggest that MMP2-cleaved collagen IV is a viable imaging target offering a specific, non-invasive strategy to visualise early and potentially reversible fibrosis (Fig. 2).

### Epigenetic and transcriptional reprogramming

A critical process during fibrosis is the differentiation of resident cardiac fibroblasts into activated fibroblasts in response to injury. This transition is governed in part by epigenetic reprogramming, which alters chromatin accessibility, transcription factor binding, and enhancer activity to orchestrate a pro-fibrotic gene expression profile [16, 42]. Genome-wide studies have revealed extensive remodelling of histone post-translational modifications during heart failure, highlighting a shift in enhancer landscapes in fibroblasts and cardiomyocytes [17]. Among the most influential regulators of chromatin remodelling are the bromodomain and extraterminal (BET) family proteins, epigenetic readers that bind to acetylated lysines on histones and facilitate the assembly of transcriptional machinery (Table 1) [18]. Of particular interest is bromodomain-containing protein 4 (BRD4), a BET protein that links extracellular stress signals such as TGF-β to gene expression, by redistributing across fibroblast enhancers in a p38 mitogen-activated protein kinase–dependent manner [10]. BRD4 promotes transcription of canonical pro-fibrotic genes, extracellular matrix components, and inflammatory mediators.

Pharmacological inhibition of BRD4 has shown therapeutic promise. The small molecule JQ1, a pan-BET inhibitor, has been shown to suppress fibroblast activation markers, reduce collagen deposition, and reverse maladaptive remodelling in murine models of pressure overload and myocardial infarction [10, 43–45]. These findings establish BRD4 not only as a therapeutic target but also as a molecular switch for fibroblast phenotype regulation. Recent advances in BET-targeted molecular imaging offer novel possibilities for monitoring chromatin remodelling noninvasively. Bai *et al.* developed a positron emitting radiotracer [11C]1 derived from a BET inhibitor [46]. This tracer showed excellent uptake and biodistribution in blood-rich organs including the heart and kidneys, with uptake blocked by pre-treatment with JQ1 confirming its specificity for BET protein. Building on this, the same group introduced ^18^F-PB006, a fluorinated positron emitting tracer designed to target the BD1 bromodomain of BET proteins with high specificity [47]. In mouse models, ^18^F-PB006 enabled quantification and visualisation of BET activity, offering a powerful new tool for assessing BRD4 occupancy and potentially fibroblast epigenetic activation.

By targeting BET proteins, positron emission tomography imaging can now visualize epigenetic activity upstream of extracellular matrix production, providing a more sensitive and temporally relevant measure of fibrotic progression. Furthermore, ^11^C-CW22, another BET positron emitting radiotracer, has been used to detect early-stage alcohol-induced tissue remodelling, with uptake correlating to BRD3/BRD4 expression in the liver and brain [47]. These studies reinforce the translational value of BET imaging in diverse tissues, and suggest potential utility in early detection of cardiac fibrosis.

### Imaging macrophages: inflammatory modulation signalling

Macrophages are key innate immune cells present in all tissues including the heart, where they perform crucial roles in homeostasis, injury response, tissue remodelling, and repair. While they are relatively sparse in the healthy myocardium, their numbers expand significantly after cardiac injury such as infarction or pressure overload [48]. These cells are highly plastic and adapt their function according to environmental cues displaying a broad spectrum of phenotypes from pro-inflammatory (M1-like) to anti-inflammatory and reparative (M2-like) profiles [49]. In the setting of cardiac fibrosis, M2-like macrophages play a central role by secreting TGF-β and platelet-derived growth factor (PDGF), both of which drive fibroblast-to-myofibroblast differentiation [50]. Moreover, macrophages themselves may directly contribute to extracellular matrix remodelling by producing matrix proteins and even transitioning into fibroblast-like cells during myocardial repair, expressing markers such as Col1A1 and Mac3 [51, 52]. This functional plasticity has made macrophages important targets for molecular imaging, particularly in the context of cardiac fibrosis where inflammation-fibrosis crosstalk determines disease trajectory. Chemokine receptors are frequently used to distinguish macrophage subsets. Notably, CCR2 positive macrophages, derived from circulating monocytes, are implicated in acute and chronic inflammation, while CCR2 negative macrophages are tissue-resident and often associated with repair [53]. Positron emitting tracers like [^64^Cu]DOTA-ECL1i, which targets CCR2, have shown promise in detecting inflammatory macrophage infiltration in models of myocardial infarction, reperfusion injury, and cardiomyocyte loss [54, 55]. [⁶⁸Ga]DOTATATE, which binds to the somatostatin receptor 2 on pro-inflammatory M1 macrophages, has identified persistent residual myocardial and bone marrow macrophage activity after myocardial infarction, revealing subclinical immune activation despite optimal medical therapy [56]. This observation was recently extended in macrophage-mediated aortic valve inflammation measured by [⁶⁸Ga]DOTATATE that predicted progression of calcification and disease severity [57].

Similarly, the [^68^Ga] pentixafor positron emitting tracer targets CXCR4, a chemokine receptor upregulated during immune activation. This tracer has been validated in both animal and human studies for imaging myocardial inflammation, particularly in infarcted regions, offering insights into disease progression and immune cell burden [12]. Despite the growing availability of such tracers, many are unable to discriminate macrophage subtypes or hybrid phenotypes involved in fibrosis. To overcome these limitations, ^64^Cu-Macrin, a polyglucose-based nanoparticle, enables broad macrophage imaging independent of surface receptor expression. Macrin positron emission tomography has demonstrated utility across a wide range of inflammatory conditions, including myocardial infarction, sepsis, pneumonia, and atherosclerosis, by capturing macrophage density and tissue distribution *in vivo* [58]. In parallel, efforts to develop dual-target probes that can report on both macrophages and fibrosis are advancing. One example is [^68^Ga]BMV101, a dual positron emission tomography/optical probe targeting cysteine cathepsins—enzymes expressed by activated macrophages and involved in extracellular matrix degradation [59]. In preclinical models of idiopathic pulmonary fibrosis and cardiac injury, BMV101 allowed visualization of both inflammatory macrophages and matrix remodelling, providing a more comprehensive readout of fibrogenic activity.

CXCR4-targeted positron emission tomography combined with T1 mapping on cardiac magnetic resonance has been used to assess early inflammation and fibrosis in non-ischemic heart failure models. Glasenapp *et al.* demonstrated that CXCR4 hybrid positron emission tomography with cardiac magnetic resonance predicted ventricular dilatation and adverse remodelling, establishing CXCR4 as a dynamic biomarker for disease staging and treatment monitoring [60, 61]. Macrophage subsets with fibroblast-like characteristics contribute directly to collagen deposition and fibrosis, but existing tracers lack specificity to capture this phenotype. Nonetheless, the distinct transcriptional signatures of these macrophage subsets, including Col1A1 positive macrophages, offer a roadmap for developing next-generation probes that distinguish inflammatory, reparative, and profibrotic macrophages [51, 62].

The interplay between macrophages and fibroblasts suggests opportunities for dual-axis molecular imaging, where immune-targeted tracers (e.g., CCR2, CXCR4) are paired with fibroblast-targeted probes (e.g. fibroblast activation protein inhibitors). Such combinations may enable clinicians to differentiate inflammatory vs. fibrotic disease phases, identify windows for immunomodulation, and detect early signs of active remodelling before structural fibrosis becomes irreversible [63].

### Imaging activated fibroblasts

Fibroblast activation protein, a type II transmembrane serine protease, is selectively expressed on activated fibroblasts involved in tissue remodelling and fibrosis but is largely absent in quiescent fibroblasts and most normal adult tissues [64]. Initially studied in the context of tumour-associated fibroblasts, fibroblast activation protein has emerged as a highly specific marker of fibrogenic activity across organ systems including the heart, where it is upregulated in response to injury, pressure overload, and inflammation [65]. [^68^Ga] and [^18^F]-labelled inhibitors of fibroblast activation protein (FAPIs), including FAPI-04, FAPI-46 and FAPI-74, have demonstrated excellent imaging properties: they are rapidly taken up by fibroblast activation protein-expressing cells, clear efficiently from non-target tissues, have high affinity binding and specificity, and deliver low radiation doses, making them suitable for repeated use (Table 1) [66]. FAPI positron emission tomography offers a molecular snapshot of fibroblast activation, correlating directly with fibrotic activity and potentially preceding adverse remodelling.

In clinical studies, [^68^Ga] FAPI positron emission tomography has identified fibroblast activation in patients with acute and chronic fibrotic disease, including myocardial infarction, myocarditis, aortic stenosis, and heart failure with preserved ejection fraction (HFpEF), diseases in which fibroblasts play a central role [67, 68]. FAPI uptake has been consistently associated with metrics of adverse left ventricular remodelling in these diseases including left ventricular ejection fraction, mass and end-diastolic volume, high sensitivity cardiac troponin I and NT-pro B-type natriuretic peptide concentrations [28, 69].

FAP expression coincides with active myofibroblast differentiation and matrix remodeling, thus its presence can discriminate between early, reversible fibrotic states and established, replacement fibrosis [28, 29, 64]. FAP has therefore also emerged as a therapeutic target. Aghajanian *et al*. demonstrated that engineered chimeric antigen receptors (CAR)-T cells targeting a model fibroblast antigen could reduce cardiac fibrosis and improve ventricular function in mice subjected to angiotensin II and phenylephrine-induced pressure overload [65, 70]. In a landmark study, Rurik and colleagues delivered mRNA encoding a FAP-specific CAR using lipid nanoparticles, effectively generating in vivo CAR-T cells that homed to activated cardiac fibroblasts and mitigated fibrosis in a murine heart failure model [71]. These findings open a promising therapeutic avenue that could be synergistically monitored or guided by molecular FAPI positron emission tomography.

### Lysyl oxidase and extracellular matrix cross-linking

As fibrosis matures, the extracellular matrix becomes mechanically reinforced through collagen cross-linking, primarily via the activity of lysyl oxidase (LOX) and its isoenzymes. This process increases matrix stiffness, impairs ventricular compliance, and limits reverse remodeling [26, 27]. Targeting LOX activity may therefore offer insight into fibrosis chronicity and tissue reversibility. Imaging agents such as ^18^F-fluoroallylamine, which bind LOX-derived cross-links, have shown promise in animal models and are under development for human use [72–74]. LOX imaging may also guide the application of anti-fibrotic therapies aimed at extracellular matrix flexibility [25].

### Metabolic imaging of fibroblast function

Activated fibroblasts exhibit metabolic reprogramming, shifting from oxidative phosphorylation to glycolysis, known as the Warburg effect [75–77]. [^18^F]fluorodeoxyglucose (FDG) positron emission tomography may detect this metabolic shift in fibroblast-rich regions [78]. However, specificity remains a challenge, and newer agents targeting glutamine metabolism or lactate export are in preclinical development for improved fibroblast targeting [12].

These imaging targets reflect a spectrum of biological processes: initiation for MMP activity, propagation for FAP, immune signalling, stabilisation for LOX, collagen cross-linking and persistence for epigenetic regulation. Future directions will benefit from combining these targets in multi-parametric or activatable probes, enabling precise molecular characterisation of fibrotic stage and activity.

## Multiplex and phenotype-driven imaging

**Fig. 3:**
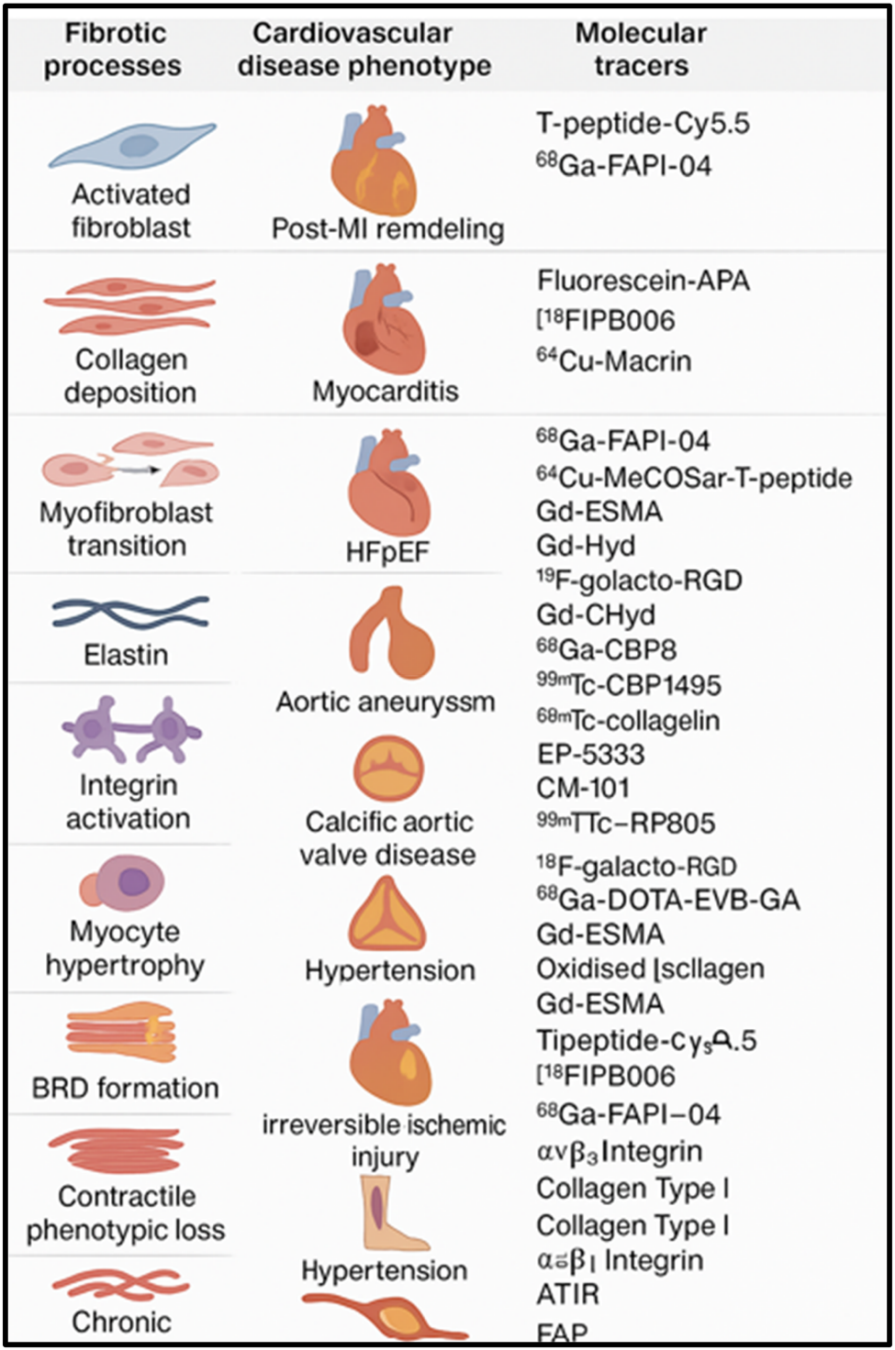
Fibrotic processes, cardiovascular disease phenotypes, and corresponding molecular imaging tracers. Mechanisms underlying cardiac fibrosis such as activated fibroblasts, elastin degradation, oxidative stress, and immune cell activation are mapped to relevant cardiovascular disease phenotypes, including pericarditis, HFpEF, aortic aneurysm, and myocardial infarction. Corresponding molecular tracers are listed, highlighting both targeted imaging agents (e.g., FAP, MMPs, TSPO) and modality-specific probes (e.g., Gd-based, ^68^Ga, ^18^F, ^99^mTc) used across clinical and preclinical platforms for in vivo assessment of fibrosis progression and therapeutic response

Technological advancements may facilitate multiplex imaging which captures the biological complexity and dynamic trajectory of fibrotic remodeling using multi-tracer protocols, bimodal probes, or spectrally distinct agents [79]. This is likely to be important because as discussed, fibrosis arises from an interdependent network of processes including fibroblast activation, extracellular matrix turnover, immune signalling, epigenetic regulation, and mechanical stress responses [1]. For example, while FAP positron emission tomography accurately localises and quantifies fibroblast activation, it cannot differentiate fibroblast subtypes, nor assess concurrent immune activity or epigenetic modulation [80, 81]. Multiplex and phenotype-driven strategies, capable of identifying multiple targets across time and space within a single biological system, are therefore of growing interest [82] (Fig. 3).

### Activatable probes, ‘omics integration, and systems-level platforms

A multiplex molecular imaging approach enabling the simultaneous or sequential detection of multiple biological processes may visualise early remodelling, map immune cell infiltration (CCR2, CXCR4) alongside fibroblast phenotype and epigenetic activity (e.g., BRD4), and track matrix crosslinking and tissue stiffening via LOX-targeted tracers [83]. For instance, a conceptual dual-tracer strategy, combining short-lived [⁶⁸Ga]FAPI to image early fibroblast activation with a longer-acting [^18^F]labeled LOX-targeted tracer for extracellular matrix cross-linking could theoretically visualise distinct fibrotic phases [83].

Activatable (“smart”) probes emit a signal only upon biological activation, including enzymatic cleavage, redox changes, or acidic pH [84–87]. Protease-activatable positron emission tomography and fluorescent agents remain “off” until cleaved by MMPs or cathepsins [88], enabling temporal tracking of protease activity *in vivo* [89]. pH-sensitive nanoparticles preferentially accumulate in inflamed, acidic myocardium, while reactive oxygen species–sensitive tracers identify oxidative stress by reacting with reactive oxygen and nitrogen species [90] in regions of fibroblast senescence or macrophage activation [91]. These platforms reduce background noise and enhance detection of transient, low-abundance signals in early fibrosis or borderline remodelling.

Advances in bioinformatics have refined imaging target selection [92]. Single-cell RNA sequencing and spatial transcriptomics in murine and human myocardium reveal fibroblast subtypes co-expressing FAP, PDGFRα, BRD4, and immune-modulatory genes [93, 94]. Temporal changes in mRNA and protein levels of proteases, as well as inflammatory cytokines, can now be imaged [89]. These insights enable the design of multi-epitope tracers or radiotracer cocktails likely to reflect clinically meaningful fibrotic phenotypes. Additionally, mining proteomic and matrisome databases can identify target clusters enriched in progressive fibrosis, supporting data-driven molecular stratification [92, 95–97]. Given that cardiac fibrosis evolves through acute inflammation, fibroblast activation, extracellular matrix expansion, and ultimately either resolution or irreversible scarring, longitudinal imaging with dynamic tracers may visualise biological trajectories rather than static disease [83].

Imaging hardware is equally critical as molecular tracers for enabling multi-target imaging. Total-body positron emission tomography systems with extended axial fields of view allow simultaneous visualisation of cardiac, vascular, renal, and haematopoietic compartments, facilitating assessment of systemic fibrotic processes [98, 99]. Increased detector coverage enhances sensitivity and resolution, supports whole-body kinetic modelling, and permits lower radiation doses [100], enabling multitracer or multi–time point studies. However, added complexity introduces signal overlap and demands advanced kinetic modelling. Regulatory pathways remain stringent for combination tracers [101], though machine learning and ‘omics-guided probe design may mitigate these challenges [102, 103].

## Disease phenotyping and risk stratification

Although left ventricular ejection fraction has historically served as the defining metric in classifying heart failure severity, a decrease in systolic function reflects late-stage functional impairment and fails to capture upstream molecular heterogeneity driving early disease. For example, heart failure with preserved or reduced ejection fraction may exhibit overlapping molecular profiles, whilst two patients with similar ejection fractions can have profoundly different fibrotic mechanisms, prognosis, and responses to therapy [104].

**Fig. 4:**
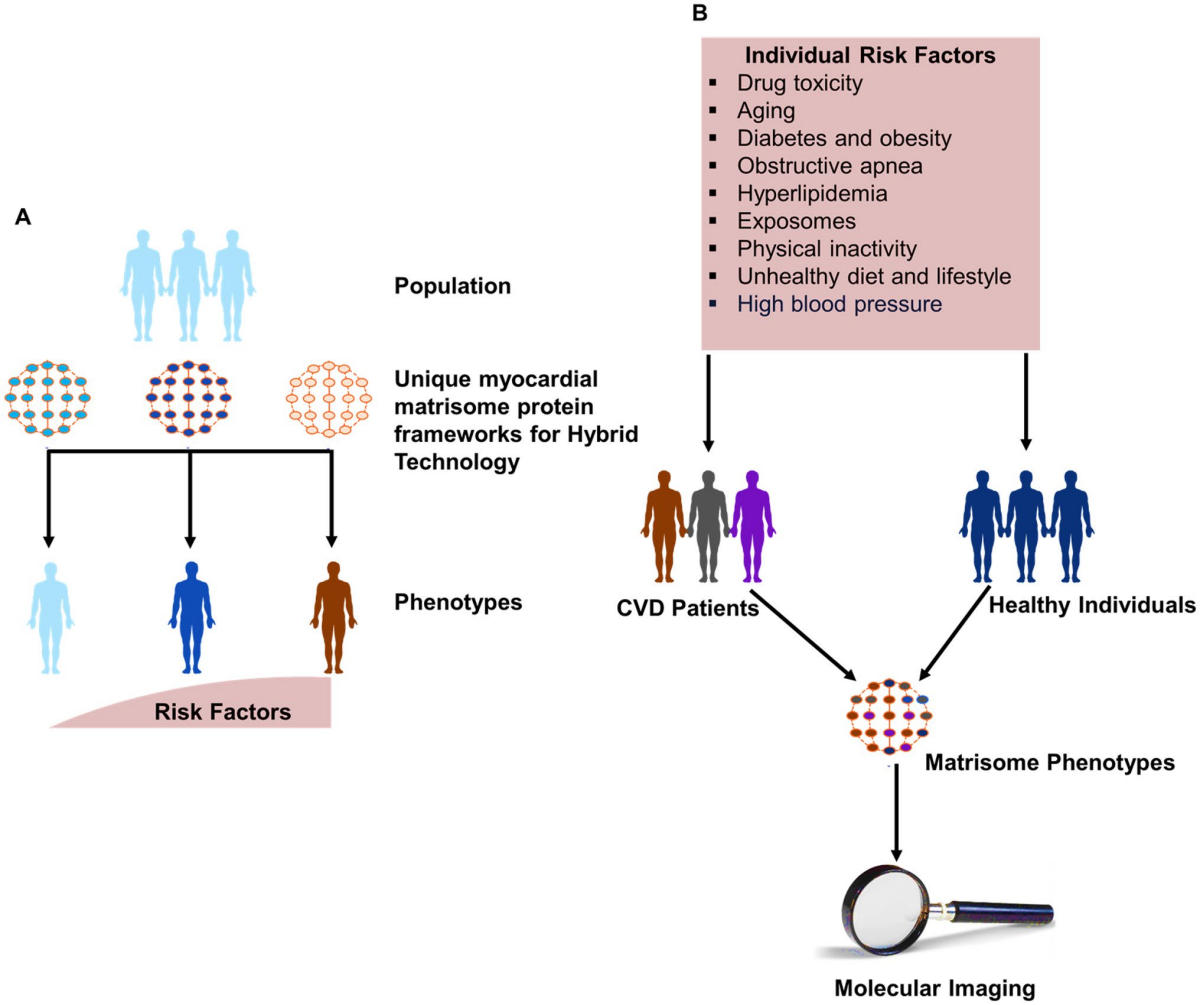
The future approach to molecular imaging in cardiac fibrosis. Molecular Imaging in specific disease and phenotype (**A**), will be facilitated by different unique molecular determinants and targets shared in a population of individuals with a disease, characterising different phenotypes. This may be indicated as risk factors, depending on the phenotypes, that will be validated to support molecular imaging of the different risk factors for at-risk individuals (**B**)

Molecular imaging may allow for more specific fibrosis endotyping [105, 106]. FAPI-based positron emission tomography, for example, has shown that some patients with heart failure exhibit intense fibroblast activation despite preserved systolic function and minimal late gadolinium enhancement on cardiac magnetic resonance [107]. Conversely, some patients with heart failure and reduced ejection fraction may not demonstrate active fibrosis on positron emission tomography, suggesting stabilisation or metabolically "burnt-out" myocardium [108–110]. This functional divergence, invisible to echocardiography or cardiac magnetic resonance, creates an opportunity to tailor therapy based on fibrotic phenotype rather than contractility alone. Imaging-defined endotypes, specific pathophysiological traits including, genetic variations or protein expression patterns associated with specific types of fibrosis can stratify patients into therapeutic categories with greater fidelity than ejection fraction thresholds. By interrogating the molecular underpinnings of extracellular matric turnover, fibroblast heterogeneity, immune-fibrotic crosstalk, and signalling pathway activation, this approach supports high-resolution phenotyping of fibrotic disease (Fig. 4).

### Risk stratification and disease trajectory

**Fig. 5:**
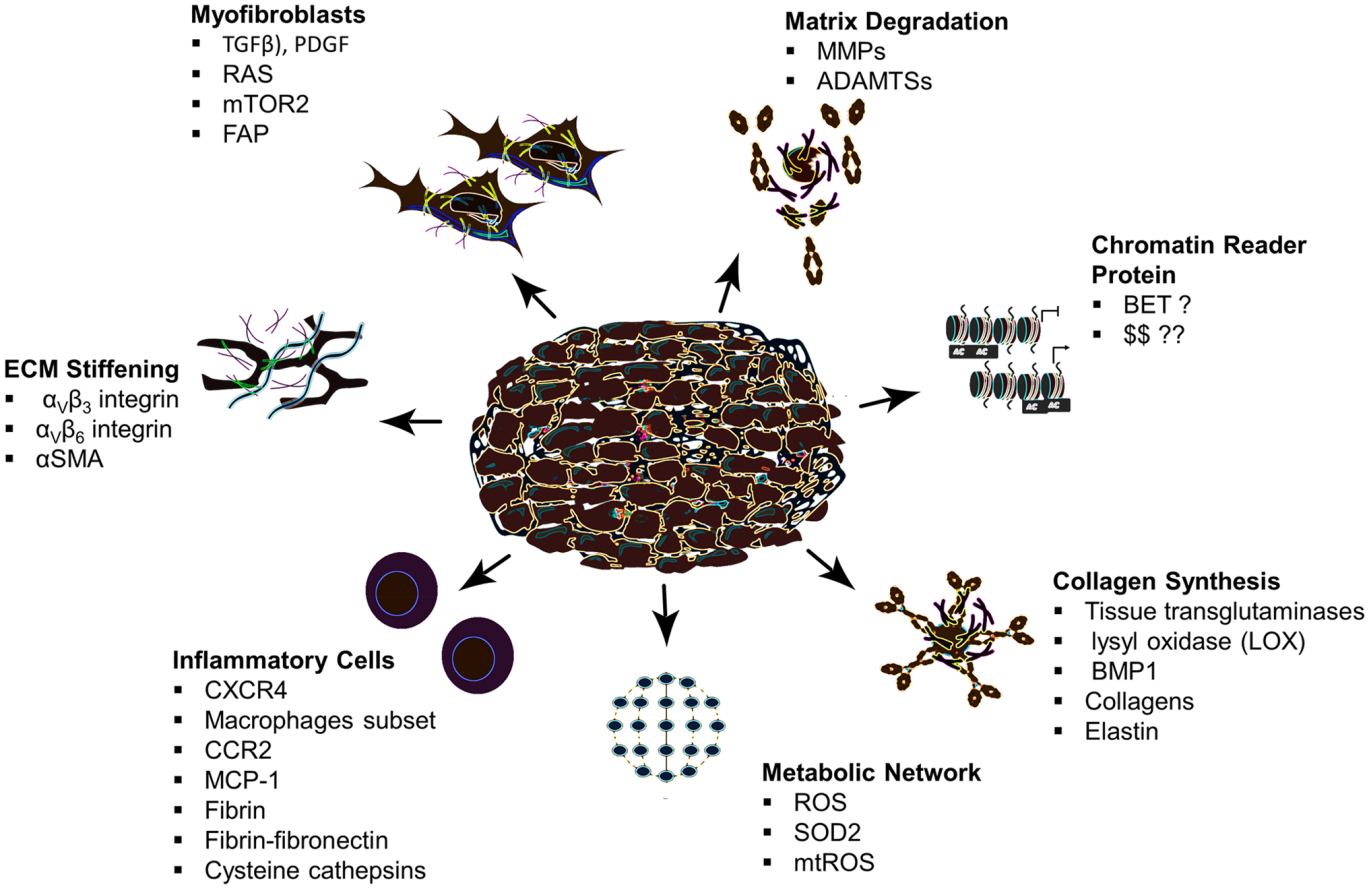
Molecular imaging targets of the extracellular matrix. Targets originate from different ECM expansion processes, during pathological cardiac remodelling. These processes include ECM turnover, fibroblast accumulation and activation, and excessive deposition of ECM proteins, ECM stiffening and contractile dysfunction, inflammation and decreased nutrient availability, epigenetic and metabolic remodelling. Targets are listed under each process. MMP, matrix metalloproteinase; ADAMTS, a disintegrin and metalloproteinase with thrombospondin motifs; TGF-β, transforming growth factor β; PDGF, platelet-derived growth factor; FGF, fibroblast growth factor; mTOR2, mammalian target of rapamycin complex 2; BMP1, bone morphogenetic protein-1; ROS, reactive oxygen species; SOD2, superoxide dismutase 2; mtROS mitochondrial-derived reactive oxygen species; BET, Bromodomain and extraterminal; MCP1, Monocyte Chemoattractant Protein-1

The burden, type, and biological state of myocardial fibrosis are powerful determinants of adverse cardiovascular outcomes, including ventricular arrhythmia and progression to heart failure. Molecular imaging is capable of capturing a dynamic risk trajectory encoded in active fibrogenesis (Fig. 4B), and may predict future events [111]. In patients with genetic cardiomyopathies, such as those with titin or lamin mutations, FAPI positron emission tomography has revealed subclinical fibrosis activity in hearts that appear structurally normal by conventional imaging, suggesting the existence of a window for early intervention before the phenotype develops [112]. Similarly, in systemic sclerosis autoimmune myocarditis, elevated uptake of FAPI and CCR2-targeted tracers has been linked to progression toward chronic cardiomyopathy, even in patients with minimal late gadolinium enhancement or modest biomarker elevations [54, 55, 113]. These tools allow clinicians to stratify patients based on molecular vulnerability (Fig. 5), enabling more refined risk assessment.

### Guiding therapeutic intervention

One of the most promising roles for molecular imaging is as a pharmacodynamic biomarker, enabling real-time, non-invasive quantification of response to therapy. Unlike conventional surrogate clinical trial endpoints such as serum NT-pro B-type natriuretic peptide concentrations and left ventricular ejection fraction, molecular imaging directly visualises the cellular and molecular pathways being targeted. ^18^F-Galacto-RGD multiparametric hybrid positron emission tomography with magnetic resonance for example has emerged as a useful early-phase tool to confirm fibroblast engagement, guide patient selection, and monitor dose–response relationships and angiogenesis after myocardial infarction [114, 115]. Trials evaluating agents such as transcriptional co-activator disruptors, or anti-fibrotic monoclonal antibodies stand to benefit from incorporating imaging endpoints that can distinguish true biological response. This is rooted in the need to accurately assess whether an experimental therapy is genuinely modifying disease biology, particularly in the context of fibrosis which progresses slowly and can be patchy or heterogeneous across the myocardium. Regulatory bodies also increasingly favour mechanistic biomarkers as endpoints in early-phase trials.

Molecular imaging may also have roles in evaluating patient selection and response to treatment for device therapy and procedural interventions [116, 117]. For example, identifying viable myocardial regions with active fibrosis may enhance lead placement and improve outcomes following cardiac resynchronisation therapy [118]. Similarly, identification of fibroblast-rich zones with active remodelling which may represent arrhythmogenic substrates could influence ventricular tachycardia ablation strategies [119, 120]. Serial imaging also supports longitudinal monitoring [121, 122], offering a sensitive measure of therapeutic efficacy. A decrease in ^18^F-Galacto-RGD tracer uptake may indicate regression of fibrosis or resolution of fibroblast activation, whereas persistently elevated signals may warrant treatment intensification or modification [123].

### Personalised medicine

With a progression towards precision medicine and with emerging therapeutics, molecular imaging may facilitate personalised care [101, 124]. Advanced imaging techniques enable granular stratification of phenotypes based on fibrotic stage, cellular drivers, and matrix composition [125, 126]. For instance, patients may present with initial inflammatory activation indicated by nuclear factor kappa B signalling, progress to active tissue remodelling marked by TGF-β and SMAD pathway engagement, and ultimately reach advanced fibrotic stabilisation characterised by myofibroblast persistence and extracellular matrix cross-linking. Similarly, fibrosis may be driven by distinct cellular contributors. such as platelet-derived growth factor receptor alpha, immune cell infiltration, or endothelial-to-mesenchymal transition, each of which may respond differently to therapy. The matrix itself also carries diagnostic information, with measurable differences in cross-linking density (LOX activity), collagen isoform expression, and protease activity [127, 128] Matreotyping using imaging and transcriptomic signatures [129] may define actionable subtypes of fibrosis across cardiac pathologies [130]. For example, heart failure with preserved ejection fraction driven by vascular rarefaction may exhibit a distinct matreotype from fibrosis secondary to pressure overload or post-infarction remodelling, and these pathophysiological variants can now be differentiated through molecular imaging [131]. This could therefore guide the application of anti-fibrotic agents, anti-inflammatory therapeutics, anti-diabetic therapies on myocardial metabolism [132, 133] or device-based interventions tailored to a patient’s molecular pathology [134].

## Future directions

### Matreotype-based classification of myocardial fibrosis

The cardiac extracellular matrix is biologically active with a molecular profile that reflects cellular activity, biomechanical stress signalling, metabolic shifts, and the directionality of tissue remodelling (Fig. 5) In a framework analogous to oncology, where tumour subtypes are now categorised based on transcriptomic and proteomic signatures [135–137], we propose that cardiac fibrosis be similarly reclassified into distinct matreotypes, such as inflammatory, metabolic, cross-linked, or senescent matreotypes based on molecular signatures. These classifications can emerge from a comprehensive integration of imaging biomarkers (such as FAP, LOX, BRD4, and MMP activity), 'omics-defined extracellular matrix characteristics including collagen isoform composition and matricellular protein expression, and fibroblast transcriptional phenotypes, as indicated by markers like Postn, Thy1 sub-lining fibroblasts, fibroblast-secreted frizzled-related protein 2 (Sfrp2), and Brd4. This would enable stratification of fibrotic states, distinguishing between active, potentially reversible remodelling and quiescent or burned out fibrosis. Moreover, matreotype classification would enhance the translatability of preclinical studies by aligning animal models to human fibrotic subtypes with greater biological fidelity [138].

### Integrating molecular imaging with multi-omics platforms

To develop and validate matreotype-based imaging tools, spatial transcriptomics, single-cell RNA sequencing, extracellular matrix proteomics, and epigenomic profiling should be integrated into the radiotracer development pipeline. Recent scRNA-seq datasets have revealed dozens of fibroblast subsets across various stages of injury and repair in both mouse and human hearts [139], providing a blueprint for identifying cell-surface proteins, secreted enzymes, or matrisome components that could serve as viable imaging targets [128]. Spatial ‘omics technologies have potential by allowing precise localisation of specific transcripts and proteins within tissue microenvironments, which can be directly correlated with imaging signals. For instance, regions demonstrating elevated FAPI uptake [140] may correspond with fibroblast clusters expressing Postn, interleukin-11, and BRD4, while areas of high LOX activity could be associated with senescent, epigenetically reprogrammed fibroblasts characteristic of aged or hypertensive myocardium. This integrative, multi-modal approach facilitates rational tracer design, prioritisation of targets, and rigorous validation of imaging specificity, ultimately creating a productive feedback loop between discovery science and imaging application.

Based on the above principles, we propose a translational roadmap for the development and clinical application of next-generation fibrosis imaging tools. This approach begins with dynamic target discovery informed not only by scRNA-seq and spatial transcriptomics [96], but also by integrative disease atlases that couple patient-derived omics with in vivo imaging signatures [141]. Here, matreotype-defined fibrosis states, classified by extracellular matrix composition, fibroblast phenotypes, and epigenetic activation, serve as the reference standard for both target selection and probe validation.

Preclinical validation should extend beyond static animal models, leveraging matreotype-matched platforms such as ex vivo perfused hearts and extracellular matrix-integrated organoids. These systems enable quantitative evaluation of tracer specificity, pharmacokinetics, and functional readouts relevant for translation to humans. Imaging should be paired with transcriptomic validation of biopsy-matched regions, and machine learning classifiers trained to assign molecular phenotypes from imaging patterns [142]. In this paradigm, each scan provides a multi-dimensional biological fingerprint. Ultimately, this approach enables adaptive precision care, where imaging not only localises pathology but anticipates disease trajectory, guiding interventions and closing the loop between discovery, imaging, and therapy in real time (Table 1).

**Table 1:** Novel molecular radiotracers, targets and application stages

Tracer name	Molecular target	Biological pathway and targeting mechanism
*Pre-Clinical*		
T-peptide-Cy5.5 [30, 31]	Collagen IV	Binds exposed or proteolytically revealed collagen IV domains associated with extracellular matrix remodelling and structural reorganisation
[18F]PB006 [43, 46]	BRD4 (BET bromodomain)	Targets BRD4 bromodomains involved in epigenetic regulation of transcriptional programs linked to inflammation, remodelling, and cellular activation states
[^64^Cu]Macrin [58]	Pro-fibrotic macrophages	PEGylated polyglucose nanoparticle phagocytosed by metabolically active macrophages, reflecting innate immune activation and fibrotic remodelling
[^64^Cu]MeCOSar-T-peptide [41]	Collagen IV	Binds structurally altered or exposed collagen IV motifs associated with matrix remodelling and proteolytic modification
Gd-ESMA [114]	Elastin	Binds mature and nascent elastin fibres, enabling assessment of vascular structural integrity, elastin content, and remodelling
Gd-Hyd [72]	Oxidized collagen	Targets reactive aldehyde groups generated during lysyl oxidase-mediated collagen oxidation and crosslinking, indexing matrix modification
Gd-OA [73]	Oxidized collagen	Binds oxidatively modified collagen species associated with extracellular matrix crosslinking and structural remodelling
[^68^ Ga]CBP8 [20]	Collagen Type I	Binds exposed collagen type I fibrils associated with fibrotic matrix deposition and structural remodelling
*Clinical*		
FAPI Tracers ([^68^ Ga]FAPI-46 or -04, [^18^F]FAPI-74) [13, 28, 67]	Fibroblast Activation Protein	Binds fibroblast activation protein (FAP) expressed on activated fibroblasts
[^68^ Ga] DOTATATE [56, 57]	Macrophages	Binds to the somatostatin receptor 2 on M1 macrophages. Produce extracellular matrix proteins and interact with fibroblasts during repair

## Conclusion

As cardiovascular care advances toward individualised treatment, there is a growing imperative to redefine how myocardial fibrosis is classified, monitored, and therapeutically targeted [143]. By interrogating the molecular underpinnings of extracellular matrix turnover, fibroblast heterogeneity, immune-fibrotic crosstalk, and signalling pathway activation, molecular imaging supports high-resolution phenotyping of fibrotic disease. Such biologically informed classification enables risk stratification of patients not just by fibrotic burden, but by underlying disease mechanisms and trajectory. This is essential for tailoring interventions to distinct fibrotic phenotypes, predicting clinical outcomes with greater precision, and optimising enrolment and endpoint selection in therapeutic trials.

## Data Availability

There is no data available from this narrative review.

## Abbreviations

BET: Bromodomain and extraterminal
BRD4: Bromodomain-containing protein 4
CAR-T: Chimeric antigen receptors T
ECM: Extracellular matrix
FAP: Fibroblast activation protein
FAPIs: FAP inhibitors
HFpEF: Heart failure with preserved ejection fraction
LGE-CMR: Late gadolinium-enhanced cardiac magnetic resonance
LOX: Lysyl oxidase
PDGF: Platelet-derived growth factor
PET: Positron emission tomography
Post: Periostin
SPECT: Single photon emission computed tomography
TGF-β: Transforming growth factor-beta
